# Advanced liquid crystal devices for augmented reality and virtual reality displays: principles and applications

**DOI:** 10.1038/s41377-022-00851-3

**Published:** 2022-05-30

**Authors:** Kun Yin, En-Lin Hsiang, Junyu Zou, Yannanqi Li, Zhiyong Yang, Qian Yang, Po-Cheng Lai, Chih-Lung Lin, Shin-Tson Wu

**Affiliations:** College of Optics and Photonics, University of Central Florida, Orlando, FL 32816 USA

**Keywords:** Displays, Imaging and sensing

## Abstract

Liquid crystal displays (LCDs) and photonic devices play a pivotal role to augmented reality (AR) and virtual reality (VR). The recently emerging high-dynamic-range (HDR) mini-LED backlit LCDs significantly boost the image quality and brightness and reduce the power consumption for VR displays. Such a light engine is particularly attractive for compensating the optical loss of pancake structure to achieve compact and lightweight VR headsets. On the other hand, high-resolution-density, and high-brightness liquid-crystal-on-silicon (LCoS) is a promising image source for the see-through AR displays, especially under high ambient lighting conditions. Meanwhile, the high-speed LCoS spatial light modulators open a new door for holographic displays and focal surface displays. Finally, the ultrathin planar diffractive LC optical elements, such as geometric phase LC grating and lens, have found useful applications in AR and VR for enhancing resolution, widening field-of-view, suppressing chromatic aberrations, creating multiplanes to overcome the vergence-accommodation conflict, and dynamic pupil steering to achieve gaze-matched Maxwellian displays, just to name a few. The operation principles, potential applications, and future challenges of these advanced LC devices will be discussed.

## Abstract (web summary)

Advanced liquid crystal-based light engines and planar optical components play pivotal roles for systematically improving the image quality and formfactor of the augmented reality and virtual reality displays.

## Graphical abstract

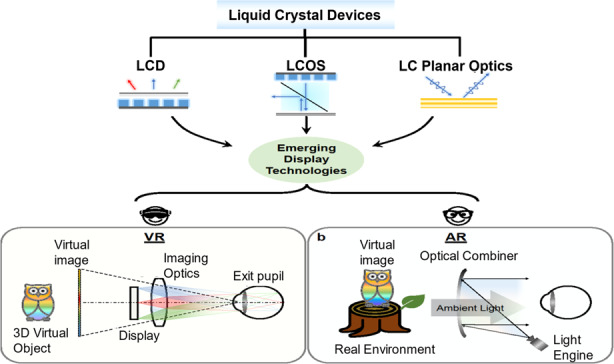

## Introduction

Display devices have become ubiquitous in our daily lives; their applications range from smartwatches, smartphones, pads, computer monitors, TVs, data projectors, to augmented reality (AR) and virtual reality (VR) headsets, just to name a few. After several decades of extensive research and development in materials and devices, and heavy investment in manufacturing technologies, liquid crystal displays (LCDs)^1–3^ and organic light-emitting diode (OLED)^4–6^ displays have become the dominant technologies. Recently, micro-LEDs^7^ and mini-LEDs^8^ are emerging; the former can offer an extremely high luminance while the latter can serve either as a locally dimmable backlight for high-dynamic-range (HDR)^9^ LCDs or as an emissive display itself. Micro-LEDs offer an ultrahigh luminance, long lifetime, and high pixel density, and are particularly attractive for the see-through AR displays^10^, especially when the ambient light is strong. Nevertheless, the biggest challenge for micro-LED to overcome is the mass transfer yield and defect repair^11^, which in turn affects the cost.

AR and VR have potential to revolutionize the ways we perceive and interact with digital information as promising next-generation displays. Since the 1990s, AR and VR experienced their first boom ushering in the dawn of evolution in display and information platforms^12^. Over the past decades, emerging technologies such as advanced liquid crystal (LC) materials and functional devices^13–17^ have greatly reshaped the AR/VR display systems, enabling this advanced information technology to be truly integrated into people’s lives. These new display platforms are mutually reinforcing with LC optics, resulting in many impressive, AR/VR-ready devices.

In this paper, we focus on advanced LC-based light engines and optical components for AR/VR applications. First, we review the basic structures of AR/VR displays and the key parameters with associated criteria. Then, we dive into three main categories of LC functional devices: HDR microdisplay for VR, high-resolution-density, high-brightness LCoS for AR/VR light engines, and nearly 100% light efficiency planar LC optics. On the microdisplay part, we discuss how an efficient, high brightness light engine improves the VR imaging system. For the LCoS panels, we firstly review the working principles and present the status of both amplitude and phase modulators, and then introduce various unique applications adopted for near-eye displays. For LC planar optics, the working principles, and promising solutions to the pressing challenges of AR/VR displays are discussed. These three LC functional devices cover the entire AR/VR systems from light engines, imaging optics, to various functional elements.

## AR and VR display systems

More recently, the next-generation display technologies under dedicated development are no longer limited to flat panels that are just placed in front of the audiences but aimed at revolutionizing the way of interactions between the users and their surrounding environment. At one end of the spectrum is VR display, which effectively extends the field of view (FoV), blocks the entire ambient, and offers a completely immersive virtual environment. At the other end of the spectrum is AR display, which pursues a high-quality see-through performance to enrich the real world by overlaying digital contents^12^. With the refreshing visual experiences, AR/VR displays have demonstrated the promising potential for a wide range of attractive applications, including but not limited to healthcare, education, engineering, manufacturing, and entertainment^18–20^.

**Fig. 1:**
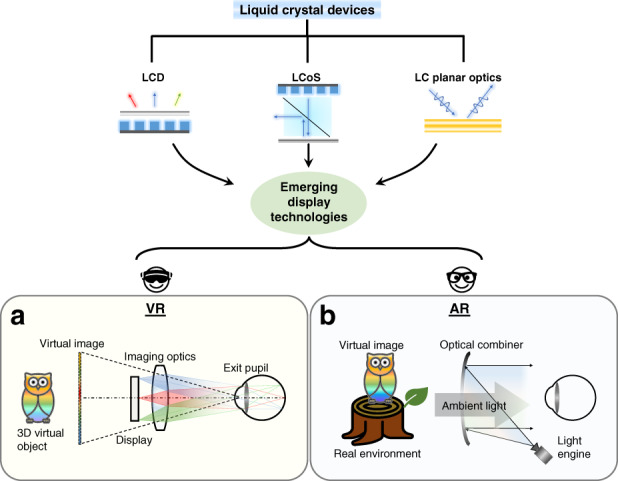
Schematic of advanced LC devices applied in AR/VR systems. Top figure illustrates advanced LC-based devices, including LCD, LCoS, and LC planar optics. These LC devices contribute to novel display technologies, including AR and VR systems. The bottom figure shows the system configurations of **a** VR and **b** AR

For VR systems, the emitting light from the display module, which is usually 2–3 inches in diagonal, generates a virtual image with an adequate FoV via the well-designed magnification lens (normally 2 inches aperture size with 35–45 mm focal length). Figure 1a depicts the schematic diagram of a VR optical system. The eyebox defines the region within which the whole image FoV can be viewed without vignetting, and its size is usually correlated with FoV due to the basic Etendue conservation. Since VR provides an immersive experience with virtual images, the 3D virtual object with depth cue is an important feature. Displaying two different images to the left eye and right eye can form the vergence cue. But the fixed image plane often mismatches with the actual depth of the intended 3D image, leading to vergence-accommodation conflict (VAC)^21,22^, which will be discussed in detail later. Perceived image resolution for VR displays can be assessed from the angular resolution and calculated by dividing the total resolution of the display panel with the FoV. To achieve human visual acuity of 1 arcminute angular resolution^23^, 60 pixels per degree (ppd) is considered a common goal. Regarding AR systems, the emitting light from the microdisplay module (usually < 1 inch) generates virtual content, which overlaps with the real world. Figure 1b depicts the generic sketch of an optical see-through AR system with an optical combiner. Throughout various optical architectures from free-space combiners^20,24^, total internal reflection (TIR) freeform combiners^25,26^, to waveguides with diffractive or reflective combiners^12^, the definition of the above parameters remains the same. But the VAC issue is more essential in AR than VR, as the image in an AR display is directly superimposed onto the real world with correct depth cues. Compared to VR with a 2–3-inch display panel, the light engine in AR is much smaller, which leads to the conflict between FoV and eyebox size, especially for a glasses-like AR system. To meet above requirements, advanced LC devices with unique optical properties and optoelectronic responses have been widely used in AR/VR displays, covering all key components from light engines to optical components.

## LCDs for VR

An LCD consists of two main parts: backlight unit and LC panel. The backlight unit provides a uniform illumination on the LC panel, and the transmittance of each pixel is controlled by a thin-film-transistor (TFT). In the past five decades, both backlight unit and LC panel have witnessed tremendous growth and significant improvement^2,27–33^. Recently, mini-LED backlight units with sufficient local dimming zones have successfully suppressed the halo effect and achieved a contrast ratio of >10^5^:1^9,34,35^. Today, TFT LCDs remain the dominant flat panel display technology, especially in TVs, tablets, and monitors, despite the strong competitions from OLED displays. The working principles and applications of LCDs as direct-view displays have been discussed in several review papers^3,8^ and will not be repeated here.

However, in a VR system, due to the emergence of many new requirements, there are some problems to directly use a traditional LCD as the light engine. To clarify these issues, in the following we will highlight the key differences between the display devices for direct-view systems and VR systems and present the challenges and potential solutions. Before diving into the details, we will briefly introduce active-matrix OLED display, which is also a strong contender for VR light engines. Compared to LCDs, the self-emissive OLED displays show an unprecedented contrast ratio, high efficiency, and fast-response time. However, the resolution density of OLED displays is not sufficient to eliminate the screen-door effect. Such a resolution limitation results from two parts: For the front plane, the traditional fine metal mesh (FMM) fabrication method can typically support organic material patterning of about 600–800 pixels per inch (PPI)^36^. For the backplane, it is necessary to squeeze the complex compensation circuit (typically, eight thin-film transistors (TFTs) and 1 capacitor) into each small subpixel (<8 µm: 3000 PPI). To overcome the above-mentioned PPI issue, the OLED-on-silicon has attracted lots of attention^37–39^. The CMOS (complementary metal-oxide semiconductor) with high electron mobility significantly reduces the circuit size. Moreover, new technologies such as laser patterned FMM^40^, silicon nitride mask^41^, photolithography method^42^, and electro-hydrodynamic printing^43^ are also emerging to help achieve high-resolution density. The other method for OLED-on-silicon to fulfill high-resolution density is to use white-OLED (WOLED) with color filters. However, color crosstalk, high driving voltage, and low optical efficiency are the key drawbacks. To address these problems, WOLEDs with microlens arrays^44^ and tandem structures^45^ have been proposed. Moreover, the high cost of silicon wafer normally restricts the display panel size to be about one inch. According to the Etendue conservation in the VR system, such a small size display panel further limits the FoV to be below 100°. Therefore, the competition between OLEDs and LCDs has extended from traditional direct-view displays to AR/VR headsets. In this review, we will focus on LCDs.

**Fig. 2:**
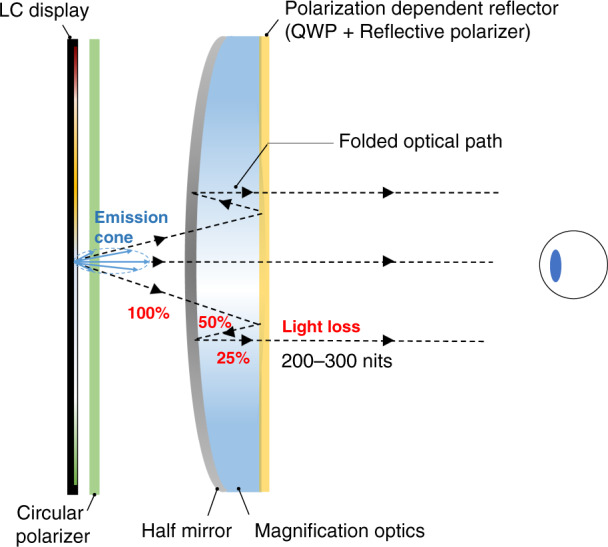
The VR systems based on pancake optics typically consist of a display panel, circular polarizer, half mirror, magnifying lens, quarter-wave plate, and reflective polarizer. Light from the display panel has a folded optical path to reduce the system thickness, which in turn helps improve the center of gravity of the VR headset

In a VR headset, an enlarged virtual image is formed in front of the user through a magnifying lens, resulting in an immersive experience. Such a projection process demands the display panel to have a much higher resolution density than that of a direct-view display. The visual acuity of the human vision system is 1 arcmin^46^, then a 6 K resolution display is typically required to support a 100° FoV VR headset^47^. To achieve a compact form factor, such a 6 K × 6 K resolution needs to be assembled on a 2–3-inch panel, resulting in a 2000 PPI of the microdisplay panel. Moreover, the motion blur becomes more severe and obvious for a near-eye system with fast eye moving^18^, especially during a video game^48^. Another major difference between traditional direct-view display and VR headset is the viewing condition. For a VR headset, the housing blocks the surrounding illumination, which significantly reduces the ambient light effect. Based on the dark adaptation of human visual system, a display with about 200–300 nits is bright enough^49^. In contrast, a TV usually requires 1000 nits. Additionally, the head-mounted devices require a compact form factor and lightweight for comfortable wearing. Recently, the folded optical path based on polarization selectivity has been widely applied in VR systems to reduce the volume size^50,51^. This thin and light design by using folded optical path is also known as pancake optics. In such a pancake system (Fig. 2), due to the light loss caused by the multiple partial reflections, the optical efficiency is reduced about four times^50^. To achieve the same brightness (200–300 nits) as a normal VR display, the required brightness from the display module should be boosted by four times^20^, which is around 800–1200 nits. Fortunately, the fixed viewing position in a VR system provides an opportunity to achieve higher efficiency by concentrating the emitted light into the eyebox area^52^.

Based on the above-mentioned requirements, there are several challenges when using LCDs as light engines in VR headsets. The first challenge is the reduced aperture ratio resulting from high-resolution density^53^. LCD panels with 100 PPI (TVs), 300 PPI (laptops), 600 PPI (smartphones), and 1500 PPI (VR light engine) have aperture ratios of 95%, 86%, 73–38%, respectively. The smaller aperture ratio reduces the optical efficiency, which in turns increases the power consumption and thermal effect of the VR headset. To mitigate the motion artifacts in VR headsets, display with high frame rate (≥120 Hz) and low duty ratio (≤20%) are preferred^54,55^. Therefore, the LC response time should be fast enough to support the high frame rate and low duty ratio^56–62^. To achieve a response time faster than 3 ms, the standing layer approach (also known as dead zones) has been demonstrated. The existence of dead zones helps shorten the response time, but the tradeoff is decreased transmittance^61^.

Overall, to achieve high-resolution density and fast-response time, LCDs for VR headsets suffer from low transmittance caused by small aperture ratios and disclination lines. If we further consider the absorption losses from color filters and polarizers, the total light transmittance of the LC panel for VR headsets is merely about 2%. Details are listed in Table 1. Therefore, how to enhance the optical efficiency of VR display panels is critically important.

**Table 1:** Total light transmission (TLT) calculation of an LC panel for VR headsets

Factors	Backlight emission	Aperture ratio	Two polarizers	Color filters	Dead zone coverage	Total
values	80–95%^a^	38%^b^	35%	33%	50%	
Calculation formula	95% × 38% × 35% × 33% × 50%					2%

^a^Depending on the LC operation mode and cell gap design employed

^b^1500-PPI condition

## Efficiency enhancement in VR

**Fig. 3:**
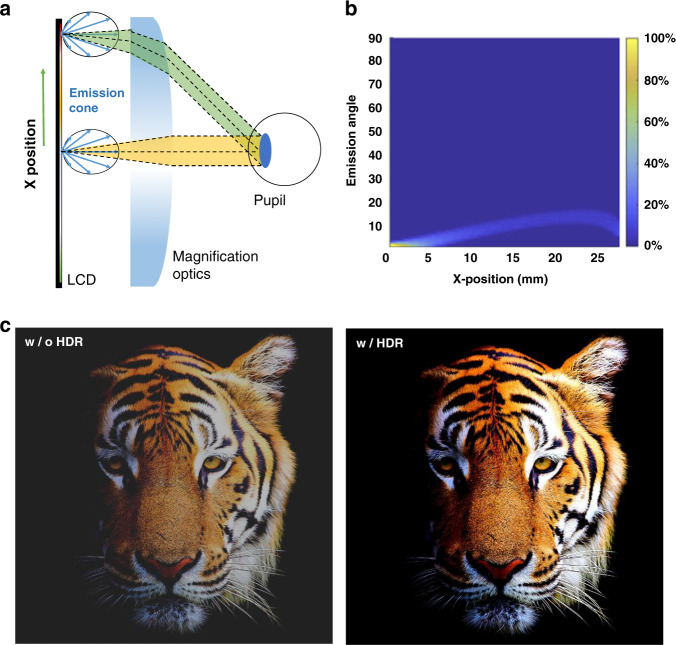
Directional backlight displays. **a** Schematic of Fresnel lens in a normal VR system. **b** Simulated light collection efficiency of different pixel positions. **c** Image comparison with and without local dimming. **b** ref. ^63^. with permission from The Optical Society, and **c** ref. ^53^ with permission from John Wiley and Sons

In this section, we will discuss how to improve LCD’s optical efficiency for VR headsets. The total optical efficiency of a VR system consists of two main components: one is the panel efficiency, and another is the imaging optics. The panel efficiency has been clearly discussed above (Table 1), here we will focus on improving the optical system efficiency using a directional LCD backlight. For a typical Lambertian display, only a small portion of the light can be received by the viewing eyebox^63^ (Fig. 3a, green and yellow cones), and the rest is wasted^64^. The system consists of an LCD, a Fresnel lens with focal length ~35 mm, and a circular receiver with a diameter of 4 mm (pupil size). The eye relief is about 15 mm, and the panel is placed on the back focal point of the Fresnel lens. The relationship between the emission angle (*y*-axis) and the light efficiency is plotted in Fig. 3b, which indicates that only a small portion of the emitted light can be received by the viewer’s eye. Therefore, a directional backlight is preferred to boost the optical efficiency of VR systems. Due to the limited volume size in a VR system, many thin and compact designs for directional backlight have been achieved by applying nanometer-sized grating, microlens arrays, micro-pyramid films, or reflective microstructures^65–70^.

The implementation of directional backlighting can significantly improve the overall optical efficiency but will also cause severe vignetting effects. As illustrated in the green cone of Fig. 3a, the chief ray of the emission cones is mismatched with the receiving angle of the eyebox. Figure 3b shows the optical efficiency at different pixel positions (*x*-axis). Only when the chief ray of the emission angle matches the receiving angle, maximum efficiency can be reached^63^. Therefore, to spatially control the direction of the emission angle is crucial to avoid vignetting while maintaining a high efficiency. To address this issue, functional optical films can be added to spatially match the radiation pattern of the display panel to the receiving cone of the VR optical system. Furthermore, Peng et al.^71^ found that the spatial radiation-pattern modulator can mitigate the ghost image in an VR system. Using a directional backlight, when the pixel position is far away from the center, the ratio of ghost to signal energy increases. The spatial radiation-pattern modulator can modulate the local radiation of each pixel and avoid receiving the ghost image. As a result, the ratio of ghost to signal energy decreases.

Aside from optical efficiency improvement, adding functional films with intensity modulation can help to enhance the VR image quality. Generally, an LCD’s contrast ratio ranges from 2500:1 (FFS mode) to 5000:1 (MVA mode). Such a grayish dark state severely degrades the user’s immersive experience. Recently, spatial intensity modulation is realized by the zoned mini-LED backlight. Depending on the displayed image, such a locally dimmable backlight can boost the LEDs in bright image areas and dim them in dark areas^72^. The mini-LED backlight technology is applied in the VR light engine to display HDR images and provide a better immersive experience. Figure 3c shows the image quality comparison of images with and without local dimming functions (2.16 inch LCD module with the 1411 PPI and 2304 dimming zones)^53^. Wu et al.^73^ packed thousands of mini-LEDs into a 2.6-inch backlight, and the brightness of each LED is modulated according to the image content. However, a notorious visual artifact, called halo effect, in the mini-LED backlit LCDs could occur, depending on the zone number, native LC contrast ratio, image content, ambient light, and viewing angle^9,35,74^. From the perspective of backlight unit, to mitigate the halo effect, it is required that the light distribution of each local dimming zone is uniform, and the light crosstalk between adjacent zones is minimized. Huang et al. described the desired light profile by a super-Gaussian function and discovered that good light confinement (power of super-Gaussian function about 4.5) helps to improve image quality. To achieve such a desired light profile, two approaches are often considered. One is to optimize the LED chip geometry, mount a scattering lightguide on top of the LED array, and fabricate reflective sidewalls and microstructures on the outer surface of the LED. Another approach is to laminate optical films in the backlight unit to shape the light profile before it enters the LC panel. Based on these two methods, novel mini-LED backlight systems supporting spatial light intensity modulation are proposed to prevent the light crosstalk and to maintain the luminance and color uniformity of the zones^34,75–79^. On the software side, Seetzen et al.^80^ proposed a local dimming algorithm (called maximum method algorithm^80^) to systematically optimize the LCD transmittance and spatial profile of mini-LED backlight. The brightness of the mini-LEDs in each zone is determined by the brightest pixel in the zone. Although a sufficient brightness can be maintained, the drastic brightness change per frame may cause severe flickering and image artifacts. The averaging algorithm, on the other hand, suppresses the peak brightness of each area to avoid such image artifacts, but the highlighted images may become dimmer and less vivid. In order to solve this dilemma, extensive works^81–83^ on novel local dimming algorithms to improve the image quality of mini-LED backlit LCDs have been proposed.

## Driving methods and power consumption of mini-LED backlit LCD

Previous sections have fully discussed the optical efficiency, in this section, we will briefly analyze the electrical efficiency to provide a comprehensive and systematic discussion. Due to the limited battery storage, an effective driving system is essential to lower the power consumption and the associated thermal effect of a compact VR headset. To drive the mini-LED backlight, both active matrix (AM) and passive matrix (PM) driving methods have been explored^53,73,84,85^. The AM driving method integrates the mini-LEDs with the TFT backplane to control the luminance of each zone and reduce the required number of IC chips^84^. For an AM mini-LED backlight, because the desired driving voltage is stored on the capacitor, the driving current can be kept constant for a long period. Therefore, the driving current is low while achieving the required luminance^86,87^. In addition, the TFT also consumes power as the driving current passing through. On the contrary, the PM driving method, which directly integrates the LCD panel with the mini-LEDs through printed circuit board (PCB), is a simpler way to drive the mini-LEDs^88^. The PM method drives the backlight row-by-row, and due to the lack of storage capability, the mini-LEDs in each row are only driven for a short time^89^. Therefore, to attain the same luminance as the AM driving, the PM method requires a higher current for the mini-LEDs.

**Fig. 4:**
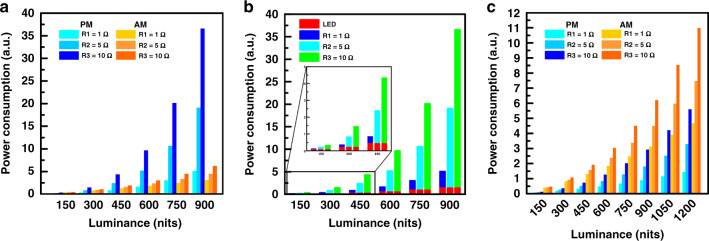
Power consumption comparison. **a** Power consumption of AM and PM driving methods with three different parasitic resistances on power line; **b** Power consumption caused by mini-LED and parasitic resistor on power line for PM driving method; **c** Power consumption of AM and PM driving with multiple scanning scheme for three different parasitic resistances on power line

To compare the power consumption between the AM and the PM driving methods, a mini-LED backlight with 48 × 48 mini-LEDs for VR displays is studied. Herein, the duty ratio of the AM and PM driving methods is set at 8.33% and 2.08%, respectively. Generally, the resistance of the parasitic resistor on the power line for AM driving is several ohms, so the resistance of 1 Ω, 5 Ω, and 10 Ω are selected for the evaluation. Figure 4a shows the simulated power consumption of AM and PM driving methods with different resistance. Overall, the PM driving consumes more power than the AM method except at low resistance (1 Ω) and luminance ≤ 600 nits. The higher power consumption originates from the larger driving current because of the shorter emission period in the PM driving method. As shown in Fig. 4b, for the PM driving method, most of the power is consumed when the large driving current passes through parasitic resistor (*P* *=* *I* *×* *R*^*2*^). Recently, to overcome the impact from short emission period, the multiple scanning scheme, which drives multiple rows at one time, is used in the PM driving method to increase the duty ratio^90^. In this work, the total rows are divided into 12 groups and the mini-LEDs of each group are driven at the same time, extending the duty ratio from 2.08% to 8.33% to reduce the required driving currents. The power consumption difference between the AM and the PM driving methods with the multiple scanning scheme are plotted in Fig. 4c. Notably, to compensate for the optical loss in the pancake configuration where the maximum transmittance drops by about four times, the display luminance should be boosted from 300 nits to 1200 nits. Within the maximum luminance of 1200 nits, the PM driving method with the multiple scanning scheme offers a lower power consumption than the AM driving method. This confirms that extending the duty ratio to reduce the driving current of mini-LEDs by the multiple scanning scheme significantly lowers the power consumption. However, more ICs are required to individually control the mini-LED in each zone, which in turn increases the cost.

## LCoS devices for AR/VR

In contrast to conventional transmissive TFT-LCDs, the reflective LCoS panel combines the electro-optic effect of LCs and high-performance silicon CMOS electronics to offer a high fill factor (>90%), high resolution, compact form factor, and high frame rate^14^. Due to the excellent light modulation ability, both amplitude and phase-only LCoS devices are important light engines for AR/VR applications^91,92^. Especially, compared to other phase modulators, such as microelectromechanical system (MEMS)^93,94^, the phase-only LCoS devices (also called spatial light modulators (SLMs)) stand out in multilevel phase modulation, low driving voltage, and comparably low cost^95^. As a result, most holographic displays for AR/VR applications are implemented with SLMs. In this section, we will first briefly describe the working principles of LCoS devices, then stress some remaining technical challenges with recent progress, and finally present their various applications in AR/VR systems.

A typical reflective LCoS device consists of a CMOS silicon backplane, pixelated aluminum reflectors, LC layer, and an indium tin oxide (ITO)-coated cover glass. The aluminum electrodes deposited onto the silicon backplane act as reflective pixel arrays. The LC layer is sandwiched between the ITO-glass substrate and the CMOS silicon backplane. When an incident light traverses the LC layer, the voltage-dependent phase retardation (for an amplitude modulator) or phase change (for a phase modulator) can be obtained^96–98^. For an amplitude modulating LCoS device in a projection system, the LED source emits an unpolarized light. Let us assume the s-polarized light (*s*-wave) is reflected, while the p-polarized light (*p*-wave) transmits through the polarizing beam splitter (PBS). The modulated *p*-wave from LCoS is reflected by the PBS onto the projection optics of the near-eye system. When a phase-only LCoS is used as an SLM with a coherent light source^99^, by spatially controlling the wavefront, the SLM can be applied in near-eye displays with holographic imaging, which will be discussed in detail later. It should be noted that the beam splitter can be replaced by other holographic optical elements (HOEs) in an off-axis configuration^92^ or waveguide geometry^100^, leading to a more compact form factor.

**Fig. 5:**
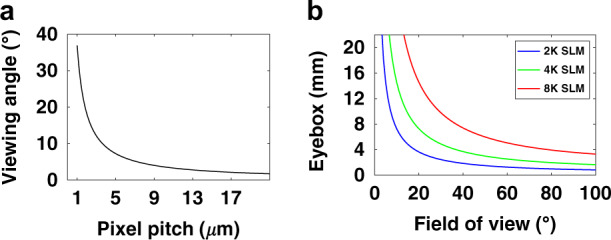
The FoV and pixel pitch in LCoS devices. **a** Relationship between the viewing angle and pixel pitch. **b** Tradeoff between the eyebox and FoV

For the LCoS based near-eye systems, the two main requirements are wide FoV and high resolution. These two factors are interrelated. In an unmagnified near-eye system, the FoV is equal to twice the maximum diffraction angle, and the eyebox is equal to the size of the employed SLM. At normal incidence, the maximum diffraction angle in a binary grating is sin^−1^ [*λ*/(2*p*_*o*_)], where *p*_*o*_ is the pixel pitch of the LCoS SLM, and *λ* is the incident wavelength. Figure 5a shows the relationship between FoV and pixel pitch at *λ* = 633 nm of the SLM. If the pixel pitch is reduced to 1 µm, the FoV can be widened to ~37^°^. The tradeoff between FoV and one-dimensional eyebox is governed by *sin*(FoV/2)·*eyebox* = *λN*/2, where *N* is the 1D number of pixels^101^. As shown in Fig. 5b, FoV can be increased for a given number of pixels at the expense of reduced eyebox size. By employing an SLM with a smaller pixel pitch and more pixels, a wider FoV can be achieved while maintaining the same eyebox size. Based on the above analysis and the requirements of AR/VR, an ultrahigh resolution of 25,400 PPI corresponding to a pixel pitch of 1 µm is critically needed.

To achieve an ultrahigh-resolution LCoS panel, the main challenges are the CMOS backplane and the fringing field effect (FFE). New LCoS foundries supporting a smaller feature size need to be developed to accommodate enough transistors in an ultrasmall pixel. Because of the limited voltage swing the CMOS backplane can support, the LC materials with a larger dielectric anisotropy are desired to reduce the required driving voltage. The layout optimization also helps achieve a smaller pixel size^102^. For the second challenge, the fringing field effect is caused by the unequal voltages on adjacent pixels. As the pixel pitch gets comparable or even smaller than the cell gap, the FFE will become much stronger, which deteriorates the LCoS performance. Currently, the state-of-the-art pixel pitch is ~3 µm for both amplitude and phase LCoS devices^103^. For an amplitude LCoS, a straightforward method to mitigate the FFE is to reduce the cell gap by employing a high Δ*n* LC material^104,105^. Besides, several other strategies have been proposed to suppress the FFE, such as optimizing the slope of pixel electrodes^106^, adopting circularly polarized light^107^, fabricating double-side electrode structures^108^, and introducing patterned pretilt angles^109^. For an SLM panel, the phase disturbance caused by the FFE could lower the diffraction efficiency and exacerbate the reconstruction error. Compared to amplitude LCoS devices, it is more challenging to mitigate the FFE of phase-only LCoS devices because of their thicker cell gap and more stringent requirements such as linearly polarized light and sharp phase edge. To achieve 1-µm-pitch phase modulators, Isomae et al.^110,111^ proposed a dielectric shield wall structure, but the device fabrication is challenging. Besides the material-based and device-based methods, model-based^112^ or algorithm-based methods^113^ have also been introduced to compensate the pixel crosstalk. Nevertheless, rigorous simulations on the LC dynamics are necessary when the pixel pitch becomes comparable or even smaller than the cell gap. Persson et al.^114^ proposed a modified algorithm to include the FFE in the hologram optimization, but the real-time operation is a main concern. Despite decades of extensive efforts, the ultrahigh-resolution phase modulators for wide FoV holographic displays are still awaiting to be developed.

## Amplitude LCoS in AR

**Fig. 6:**
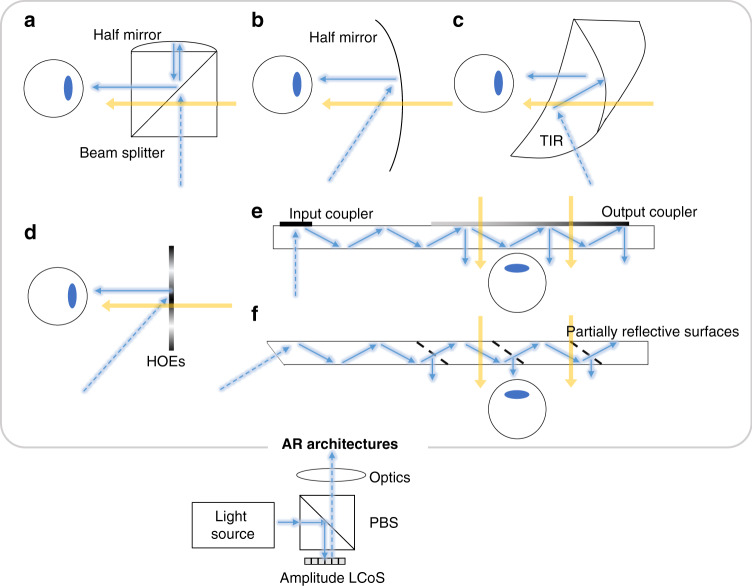
Amplitude LCoS for different AR architectures. **a** Birdbath optics with beam splitter. **b** Single freeform surface as combiner. **c** Freeform TIR prism as the combiner. **d** HOEs-based combiner with compact form factor. Waveguide AR with **e** diffractive couplers and **f** partially refractive couplers

For AR displays, several types of light engines have been developed as the image source to provide the virtual images, such as LCoS, digital light processor (DLP), OLED microdisplay (μOLED), micro-LED, and laser beam scanner (LBS)^12,115^. LCoS can provide high brightness (>50 K nits) and has come to fabrication maturity after more than 20 years of development. Companies like Sony, Himax Displays, Compound Photonics, etc., are devoted to developing LCoS based light engines with high resolution for AR applications and the total volume is only about 1 cm^3^ including the illumination optics^15,116^. As shown in Fig. 6, an amplitude LCoS has been integrated into several types of AR systems. For example, Google Glass adopts a traditional PBS as optical combiner and utilizes a Field Sequential Color (FSC) LCoS with resolution of 640 × 360 as its light engine. The combiner designs used in Google Glass are often referred to as birdbath optics (Fig. 6a). Another simpler design using a single partially reflective off-axis freeform mirror as the combiner can be seen in later products such as Meta 2^117^, achieving a large FoV (up to 90°) with the cost of form factor (Fig. 6b). Another way to fold the optical path is to use a TIR-prism (Fig. 6c). In this design, all the surfaces are freeform, which offers an excellent image quality^25^. To cancel the optical power for the transmitted environmental light, a compensator is added to the TIR prism. The whole system has a well-balanced performance between FoV, eyebox, and size. To further reduce the form factor, the prism/half mirror can be replaced by thinner and angular-sensitive HOEs (Fig. 6d) and achieve a glasses-like form factor^118,119^. However, these HOEs show a strong wavelength dependence thereby suffering severe dispersion issues. In the early days, amplitude LCoS are widely used in data projectors, where real images are projected to a scattering screen for audiences to see with a large viewing angle. With the aid of see-through in AR displays, Lee et al.^120^ proposed a projection system based on diffusive HOEs, where most ambient lights pass through the diffusers due to the narrow angular selectivity of the HOEs. One common configuration for generating such images is to modulate a collimated beam with an amplitude LCoS. Another configuration for using LCoS as a display panel is to add pinholes to a 4-*f* system, where a collimated light source is no longer required^121^.

Currently, most AR products adopt waveguide design with diffractive (Fig. 6e) or reflective (Fig. 6f) combiners due to the attractive thin form factor. Among them, HoloLens 1 and Magic Leap 1 and 2 adopt LCoS as light engines^122,123^. A newly developed AR prototype, Lumus Maximus^124^ combines a 2 K × 2 K LCoS with its partial reflective surfaces waveguide, achieving a 50° FoV and showing an impressive image quality. It is worth mentioning that LCoS itself is a reflective display operating with a polarized light, while most light sources are unpolarized, including traditional lamps and energy-efficient LEDs. To improve the light efficiency, a polarization converter can be integrated into the amplitude LCoS thereby increasing the light efficiency^125–129^.

## SLMs for AR/VR

**Fig. 7:**
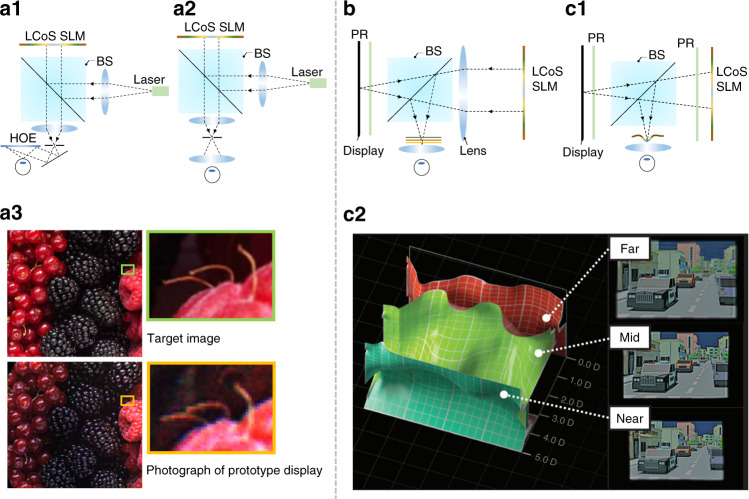
Phase-only LCoS for AR/VR systems. **a1** Schematic of phase LCoS based AR system. **a2** Schematic of phase LCoS based VR system. **a3** Image quality comparison between the target and the photography of the prototype. **b** 4 f system with a LCoS SLM. **c1** Focal surface system configuration with a LCoS SLM. **c2** The optimized focal surfaces and color images displayed across three frames. Adapted from **a3** ref. ^92^. and **c2** ref. ^137^. under the Creative Commons Attribution 4.0 License

A high-resolution LCoS SLM can directly control the properties of the illumination wavefront, allowing to support holographic view^130^, which is also referred to as computer-generated holography (CGH)^101^. The generated holographic image exhibits the natural focus-and-blur effect like a real 3D object. Such an SLM is particularly useful for AR/VR displays to provide the 3D scenes with correct depth cues. Maimone et al.^92^ demonstrated a high-quality imagery with a compact holographic view for VR and AR displays using a SLM. Based on Fresnel holographic principles, the system can integrate most of the imaging complexity into a SLM, enabling precise and spatially variant control of image focus. This control is the core for generating holograms. Figure 7a1 and a2 show the system configurations for AR and VR, respectively. Here the pitch size is 8 μm, and the active area is 15.36 × 8.64 mm^2^. Figure 7a3 shows an image quality comparison between the target and the photograph of the prototype VR display. It is encouraging to achieve holograms with SLM for near-eye displays. However, the hologram rendering requires complicated computation and is time-consuming. Another concern for further using an SLM in near-eye displays is the limited Etendue. Presently, the smallest pixel size of an SLM is ~3 µm (usually 3–12 µm), therefore the diffraction angle is generally <5° under plane-wave illumination. A typical SLM panel size is only 0.5–1 inch, as a result, the tradeoff between eyebox size and FoV needs to be balanced.

To use a LCoS SLM for AR/VR displays with an expanded Etendue, one strategy is to increase its diffraction angle by relay optics or applying spherical-wave illumination^131–133^. Chen and Chu^131^ demonstrated a 4-*f* relay system to demagnify the SLM. The imaged pixel size becomes smaller via the 4-*f* system, leading to a larger diffraction angle at the cost of a smaller display panel. Then Chen et al.^132^ used a spherical wavefront to illuminate the SLM, increasing the diffraction angle and thereby the FoV. Meanwhile, Shi et al.^133^ also demonstrated a near-eye holographic system with spherical waves for a wider FoV. Aside from above solutions, Kuo et al.^134^ developed a scattering-based Etendue expansion method, in which the outgoing coherent beam from the SLM is scattered by a static mask with randomized scattering properties. The wavefront coming from the SLM can be scattered by the mask to a larger range of angles, thus increasing the Etendue. As a result, the FoV can be extended without decreasing the eyebox size. By applying this approach, the FoV was experimentally expanded by four times in both horizontal and vertical directions. It is worth noting that the scattering masks are randomly patterned, leading to the inevitable noise of these randomly scattered lights, which will cause low fidelity problems when extending the system Etendue.

Recently, based on the scattering mask concept, Baek et al.^135^ proposed a neural Etendue expander with wide-angle and high fidelity for near-eye systems. Here, the generation of neural Etendue expander and the state of SLM were jointly trained via neural network across a natural image dataset. As a result, the trained Etendue expander possesses the benefits of scattering elements for enlarging FoV, while improving the signal-to-noise ratio (SNR). In the simulation, this approach can increase the Etendue by sixty-four times, which means a wide FoV and a large eyebox can be achieved simultaneously.

Due to the relatively high cost and limited panel size with today’s hardware, using an SLM as the light engine for AR/VR displays is not yet practical. However, SLMs can be used as an extra booster to combine with traditional display penal, such as LCD, for near-eye displays to overcome the VAC issue. Due to its phase modulation ability, an SLM can be placed in the light path as a spatially controllable lens. Cui and Gao^136^ demonstrated a multifocal system by dividing a display panel into four subpanels and optically mapping them to different depths with an SLM located at the Fourier plane of the 4-*f* system (Fig. 7b). Here the SLM presents a static phase profile, including the quadratic phases that image the subpanels to different depths. Meanwhile, Matsuda et al.^137^ also designed and demonstrated a full-color focal surface display to address the accommodation issue for near-eye displays. In this design, the SLM works as a programmable lens with spatially varying focal length, which can optically map the pixels at different spatial locations to different depths. As shown in Fig. 7c1, the focal surface display has a more compact form factor than the design reported by Cui and Gao due to the absence of the 4-*f* system. Moreover, the focal surface display can support arbitrary depth maps compared to conventional multifocal displays thereby displaying more accurate depth and blur (Fig. 7c2). Here, the burden to generate correct depth is placed on the computation, which makes real-time global optimization of phase patterns on SLM very challenging. The good news is that with the advancement of algorithms and convolutional neural networks (CNNs)^138,139^, solving this problem is just around the corner. Recently, Shi et al.^139^ demonstrated a real-time CGH synthesis pipeline with a high image quality, achieving a 60-Hz frame rate through a desktop computing unit.

## LC planar optics

In addition to modulating the image as a display or SLM, LCs exhibit some other attractive features, such as polymerization and photo-patternable characteristics^140,141^. These properties can be used to create new photonic devices, called LC planar optics or LC optical elements (LCOEs)^142–144^, which exhibit a ultrathin form factor, nearly 100% efficiency, strong polarization selectivity, and switching ability. These LCOEs show promising applications in AR/VR systems. different form light engine. In this section, we will discuss the LCOEs from transmissive and reflective aspects, respectively.

## Transmissive LC planar optics

**Fig. 8:**
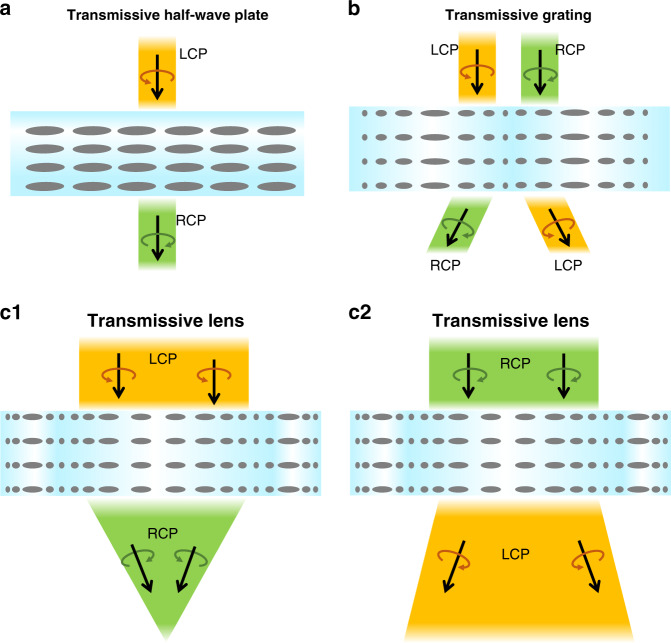
Diagrams of polarization responses of various transmissive LCOEs. **a** transmissive waveplate**, b** transmissive grating, and **c** transmissive PB lens

In the transmissive LCOEs, we will focus on the geometric phase optical elements, whose phase retardation depends on the LC alignment. This is the main reason that the transmissive type optical element can achieve sharp phase modulation within a thin layer. Being a diffractive optical element, this type of device is working in the Raman-Nath regime, which is regarded as the “thin” optical element (e.g., surface relief grating), and its diffraction efficiency can be maximized at an optimal thickness. Such a transmissive LCOE will show strong polarization selectivity and can work as a waveplate, grating, and lens, respectively, depending on the LC alignments. The polarization responses of each element are summarized in Fig. 8. In the following, we will discuss each device in detail including its working principles and promising applications in AR/VR displays.

**Fig. 9:**
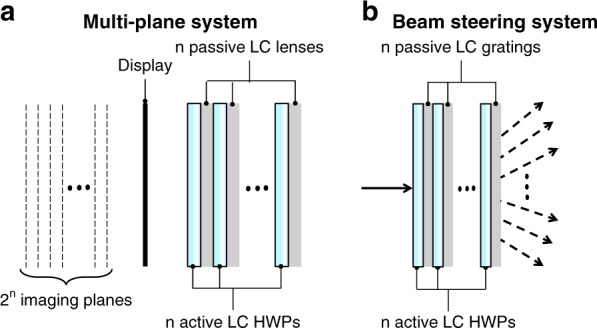
Applications of LC wave plate in AR/VR. **a** Multiplane system configuration. **b** Beam steering system configuration

Being an anisotropic optical material, LC can introduce phase retardation for a polarized light^145^, thereby it is a promising candidate for waveplates (or phase retarders). Both passive (LC polymer) and active (LC cell) waveplates can be fabricated^146,147^. The passive LC waveplate is a polymer film after UV stabilized process, which is ultrathin and light. The active LC waveplate is switchable between on- and off- states by applying a voltage. The schematic of a simple LC half-waveplate (HWP) is depicted in Fig. 8a. Lu et al.^148^ proposed a method to achieve multiplane near-eye display by using active LC waveplates, as shown in Fig. 9a. In the system, the LC-based active HWP works together with a passive LC lens. Since the LC lens is polarization dependent, switching the active HWP will rotate the polarization state, which in turn changes the optical power. If the passive LC lens is replaced by a polarization selective grating, then the focal plane modulation will turn to output beam angle shifting for beam steering, as Fig. 9b shows^149^. This compact beam steering has potential for pupil steering^150^ and foveated imaging^151^ in AR/VR systems. To have a broadband active waveplate, the simplest device is a TN cell. Moreover, we can fabricate a broadband active waveplate by using a negative-dispersion LC material to compensate for the wavelength dependent phase retardation in the visible range. Merck has developed such a negative-dispersion LC monomer (e.g., RMM 1705) for passive LC waveplates. However, for multiplexing and steering applications in the AR/VR systems with an active LC waveplate, the response time needs to be fast (~1 ms). At present, no negative-dispersion LC with such a fast-response time has been reported yet. Another approach is to build multi-twist structure^152^. According to the requirements of active driving, each twisted layer needs two ITO-glass substrates, which increases the thickness and weight of the system substantially.

An LC grating can be obtained by introducing a linearly varying phase profile to the LC waveplate. Several methods have been developed for generating LC grating patterns: one is to create a periodic electric field by making periodic electrodes^153–155^ or periodically modulating the electric field distribution^156^, and another is to provide volume/bulk/surface alignment to the LC molecules^157–164^. Currently, the most widely employed method is surface alignment with interference exposure, which can achieve a smaller grating period and smooth phase change. This method is also suitable for low-cost mass production. In the interference exposure, the grating pattern can be described as:

1 $$\left[ {\begin{array}{*{20}{c}} 1 \\ i \end{array}} \right]e^{ - ik_0x\sin \theta } + \left[ {\begin{array}{*{20}{c}} 1 \\ { - i} \end{array}} \right]e^{ik_0x\sin \theta } = 2\left[ {\begin{array}{*{20}{c}} {\cos (k_0x\sin \theta )} \\ {\sin (k_0x\sin \theta )} \end{array}} \right]$$

from Eq. (1), the grating pattern is sinusoidal linear polarization whose diffraction efficiency at normal incidence is:

2 $$\eta _0 = \cos ^2\left( {\frac{{\pi \Delta nd}}{\lambda }} \right),\eta _{ \pm 1} = \frac{{1 \mp S^{\prime}_3}}{2}\sin ^2\left( {\frac{{\pi \Delta nd}}{\lambda }} \right)$$

where $S^{\prime}_3 = S_3/S_0$ is the normalized Stokes parameter of the incident light. Due to linear phase change of the grating pattern, only the 0^th^ and ±1^st^ orders have nonzero solution, when the grating transfer matrix has the Fourier transform for the far field diffraction^165^. In other words, according to Eq. (2), when the grating thickness satisfies the half-wave condition, all the diffracted light will contribute to the ±1^st^ orders. Furthermore, if the incident light is circularly polarized, the diffraction efficiency can reach 100% in theory. Under this condition, the light propagation process can be expressed by Jones matrix:

3 $$J_{HWP}\left[ {\begin{array}{*{20}{c}} 1 \\ { \pm i} \end{array}} \right] = \left[ {\begin{array}{*{20}{c}} {\cos (2k_0x\sin \theta )} & {\sin (2k_0x\sin \theta )} \\ {\sin (2k_0x\sin \theta )} & { - \cos (2k_0x\sin \theta )} \end{array}} \right]\left[ {\begin{array}{*{20}{c}} 1 \\ { \pm i} \end{array}} \right] = \left[ {\begin{array}{*{20}{c}} 1 \\ { \mp i} \end{array}} \right]e^{ \pm i2k_0x\sin \theta }$$

as we plotted in Fig. 8b, the incident circularly polarized light will be converted to the opposite handedness with an extra phase, and this phase is also known as geometric phase or Pancharatnam-Berry (PB) phase^166^. PB phase grating has attracted lots of attention because of its nearly 100% diffraction efficiency^149^. It can be achieved with an electro-optically controlled active grating^167,168^ or a polymer-based passive grating. Moreover, such a passive PB grating can adopt dual-twist^142,169^ and multi-twist^170^ structures to achieve broadband and wide-view performance.

**Fig. 10:**
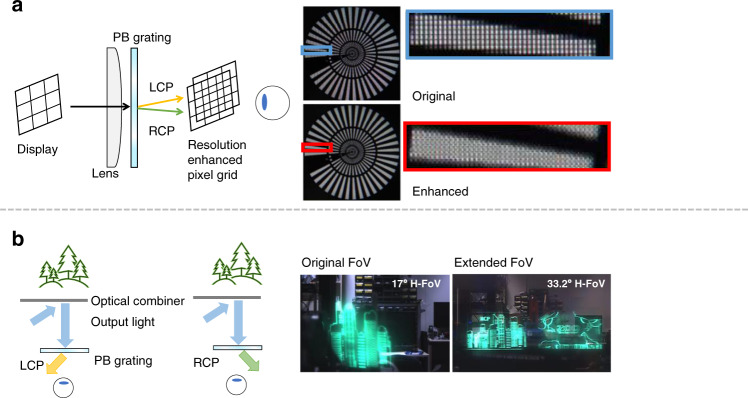
Applications of transmissive LC grating in AR/VR. **a** Resolution enhancement system for VR with experimental results. **b** Polarization multiplexing AR system for extended FoV with experimental results. H-FoV: horizontal field of view. Reprinted from **a** ref. ^171^ with permission from The Optical Society and **b** ref. ^173^ with permission from The Optical Society

For VR applications, Lee et al.^171^ used an active PB grating to enhance resolution by modulating the imaging position with time multiplexing. As a result, the original pixel grid and the shifted pixel grid appear alternatively by frames, thereby the apparent pixel density is doubled (Fig. 10a). Zhan et al.^172^ also applied a passive PB grating to realize similar pixel shifting by polarization multiplexing rather than time multiplexing. To precisely control the polarization state, a pixelated polarization rotator is added to align with the original display panel. For AR applications, Yoo et al.^173^ proposed a waveguide AR system with an extended FoV by using such a PB grating. As depicted in Fig. 10b, the output light with different circular polarization states is deflected into opposite directions. By combining images from two orthogonal polarizations (LCP and RCP), the FoV is doubled in horizontal direction. Besides, PB phase grating can also be used to enlarge the eyebox of Maxwellian system. For example, Lin et al.^174^ demonstrated a 2D pupil duplication in Maxwellian view by stacking two 1D PB gratings and one QWP together. Such a stacked 1D device can also be replaced by a 2D PB phase grating. Shi et al.^175^ proposed a multi-beam interference method to generate the 2D grating pattern, and He et al.^176^ also fabricated a PB Dammann grating using projection lithography.

An LC lens can be obtained by introducing a parabolically varying phase to the LC waveplate. The lens pattern can also be generated by electric field modulation and pattern alignment, as described in the LC grating section^177–180^. The surface alignment method enables the PB phase to generate an LC lens whose optical power is independent of the LC birefringence^181^. Similar to a PB phase grating, the PB phase based LC lens (PB lens)^161,182,183^ is also a polarization selective optical element. Its focal length depends on the LCP or RCP input (Fig. 8c). Both passive and active PB lenses can be fabricated using a polymer film or an LC cell, respectively^184^.

**Fig. 11:**
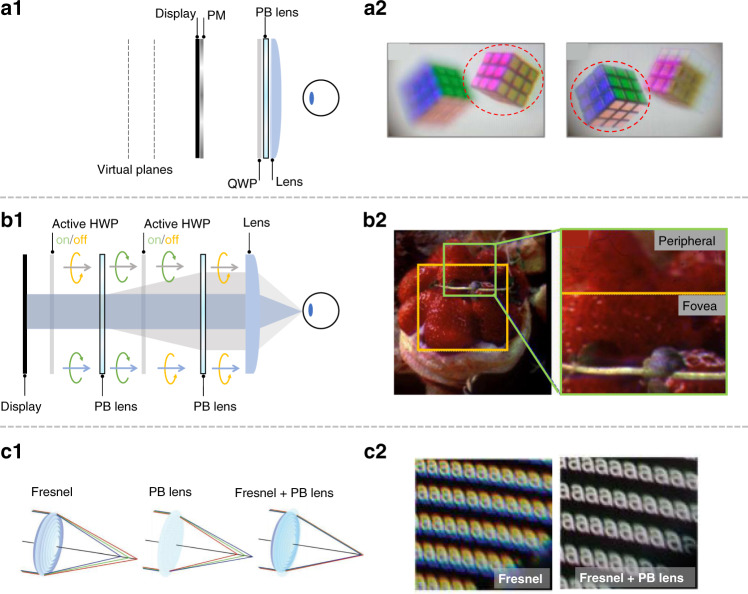
Applications of transmissive LC lens in AR/VR. **a1** Polarization multiplexing based multiplane VR system with **a2** experimental results. **b1** Foveated VR system with **b2** experimental results. **c1** Chromatic aberration correction for VR system with **c2** experimental results. Reprinted from **a2** ref. ^185^ with permission from The Optical Society, **b2** ref. ^186^ with permission from The Optical Society, **c2** ref. ^188^ with permission from John Wiley and Sons

Based on this unique polarization dependent focal length, Tan et al.^185^ realized a polarization multiplexed multiplane VR display by using a passive PB lens. The working principle of polarization multiplexing is depicted in Fig. 11a1. The system requires a pixelated polarization modulator (PM) to control the ratio between RCP and LCP, allowing independent generation of image content on two virtual planes (Fig. 11a2). Polarization multiplexing is an elegant solution for creating multiplane displays without sacrificing the frame rate, while keeping the headset lightweight for long-term wearing. However, its limitation is that there are only two orthogonal polarization states, and theoretically only two imaging planes can be generated. Yoo et al.^186^ presented a foveated display system by using two PB lenses. In this system (Fig. 11b1), the opposite focal lengths of LCP and RCP lead to two modes with high- and low-angular resolutions. By switching between LCP and RCP, both high and low-angular resolutions can be perceived with different spatial positions, thereby a foveated imaging can be generated (Fig. 11b2). The reported system has an optical efficiency of 12.5%, and both active and passive PB lenses can be used for this system. However, this approach requires a space between lenses for the beams to expand to different diameters. How to accomplish a small form factor could be challenging.

For current near-eye displays, a high optical power lens with compact form factor is required, which usually has significant chromatic aberrations^187^ near the edge field. To solve this issue, a hybrid lens (combining a passive diffractive PB lens with a refractive lens, Fig. 11c1) has been proposed. Due to the opposite dispersion, their chromatic aberrations can be compensated each other (Fig. 11c2)^188^. Besides, active PB lens is a good adaptive optics candidate for the AR/VR displays to generate light field display by switching between each polarization responses^189^. Based on recent advance in fast-response LC materials^190^, the switching time of 1–2 ms has been demonstrated in active PB lens or LC HWP. Besides, LC microlens array (MLA)^191,192^ and multi-domain LC lens also play important roles in AR/VR systems. One of the important applications of MLA is the integral imaging light field display^193^. The system can generate multiple views to reconstruct the real object light field, thereby the user can observe 3D images with both amplitude and angular information. Currently, the reported integral imaging systems use traditional glass MLA because of its good imaging quality and acceptable form factor. Potential applications of LC MLA with tunable focal length and polarization selectivity in integral imaging display systems are foreseeable^183^.

## Reflective LC planar optics

**Fig. 12:**
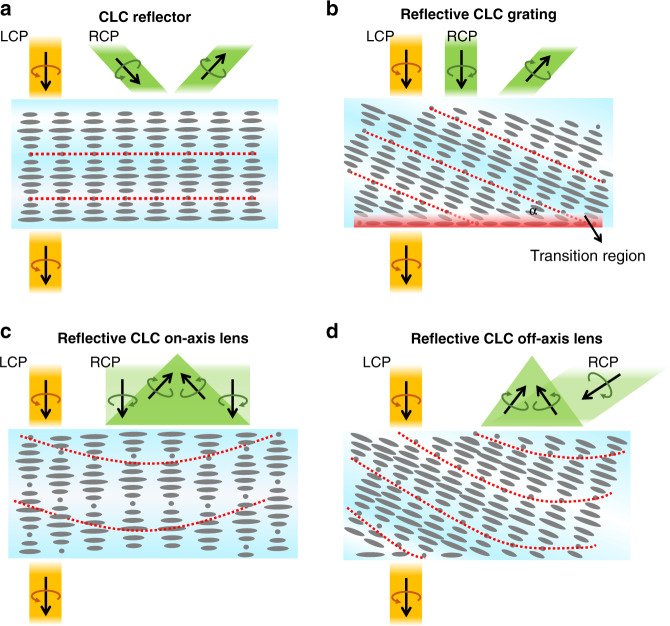
Diagrams of polarization responses of various reflective LCOEs. **a** CLC reflector, **b** reflective CLC grating**, c** reflective on-axis CLC lens, and **d** reflective of-axis CLC lens

In a reflective LCOE, the reflection mechanism is based on the Bragg reflection of the employed cholesteric liquid crystal (CLC)^194–196^. Therefore, this type of diffractive optical element is called as “thick” optical element (e.g., Bragg volume grating). The efficiency of such a reflective LCOE is closely related to the device thickness. To establish Bragg reflection, the minimal LC layer thickness is about ten pitches. Besides, the helical CLC structure can be achieved by doping some chiral dopants to a nematic LC host^197^. To accommodate a wide viewing angle and broadband, we can apply more complex structures, such as multilayers and gradient pitch^198–200^. As plotted in Fig. 12, the red dashed lines connect the short axes of the LC directors and represent the Bragg surface. Depending on different Bragg surfaces generated by the LC alignments, such a reflective LCOE exhibits strong polarization selectivity and can work as a reflector, grating, and on-/off- axis lens, respectively. The polarization responses of each element are summarized in Fig. 12.

**Fig. 13:**
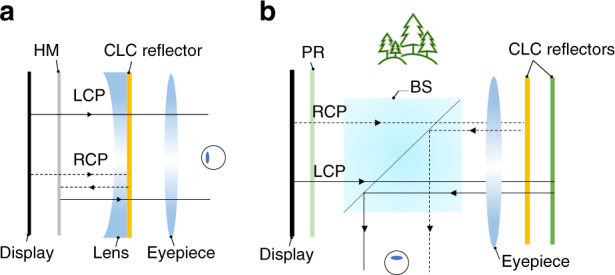
Applications of CLC reflector in AR/VR. **a** Foveated VR display; **b** Multi-depth AR display

The CLC reflector (Fig. 12a) has a simple structure but can contribute to various applications for AR/VR. In the VR pancake structure, a CLC reflector can replace the QWP and reflective polarizer to obtain a simpler structure. Furthermore, a foveated VR display can be achieved via encoding two images into orthogonal circular polarizations by using CLC reflector^201^. As shown in Fig. 13a, the images undergoing different paths experience different magnifications, and therefore the spatial resolution of the foveal image could be greatly enhanced. Leveraging the polarization selectivity, CLC reflectors can also generate two optical paths in an AR system. Chen et al.^202^ demonstrated a dual-depth AR system by using two CLC reflectors which are separately placed in sequence to produce two different diopters and optical paths, where each path corresponds to one image depth (Fig. 13b). By employing multiple CLC reflectors, more image depths can be produced.

A reflective LC grating can be obtained by introducing a linearly varying phase to the CLC reflector, called polarization volume grating (PVG). This linearly changed phase can be generated by surface alignment^203–206^. Compared to a CLC reflector, the PVG has a more complex structure, where the azo compounds on the photoalignment layer exhibit a sinusoidal pattern along the horizontal axis. Figure 12b depicts the LC distribution of the PVG structure. The Bragg surface is still the line connecting the short axes of the LC directors but with a slanted angle (α). The bottom LC directors tend to follow the sinusoidal pattern, but the helical axis of the bulk CLC is tilted (~25^o^) to minimize the free energy, therefore producing a transition region from the bottom to top. When the incident beam satisfies the Bragg condition, the diffraction angle is twice the slanted angle, and the diffraction efficiency is the highest^207^.

**Fig. 14:**
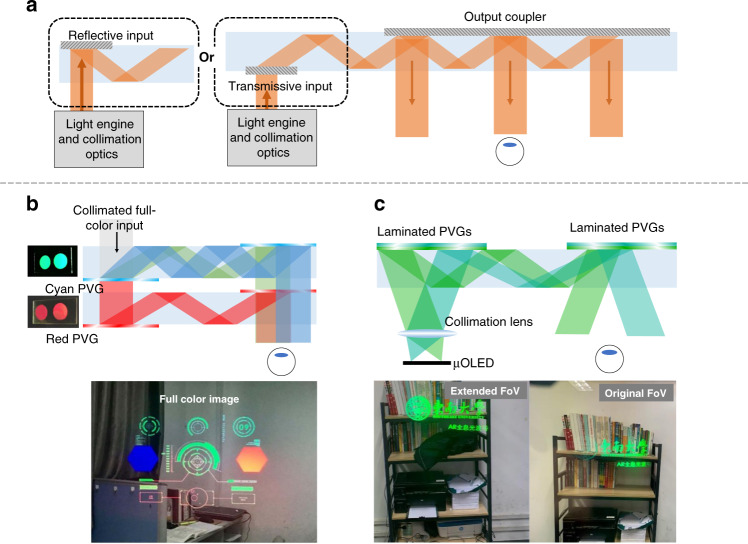
Schemes of diffractive waveguide combiners and applications of PVG in waveguide AR. **a** Working principles of waveguide AR (transmissive input coupler and reflective input coupler). **b** System configuration of full-color waveguide AR display with experimental results. **c** Enlarged-FoV waveguide AR display with experimental results. Reproduced from **b** ref. ^213^ with permission from The Optical Society. Reprinted from **c** ref. ^214^ under the Creative Commons Attribution 4.0 License

In recent years, the reflective LC diffractive couplers (e.g., PVGs) have also been widely studied and demonstrated in lab-level waveguide-based AR prototypes^208^. Before diving into the PVG-based waveguide structure, we briefly introduce the basic operation principles of the diffractive waveguide AR. As shown in Fig. 14a. the waveguide architecture has diffractive input and out-couplers to deliver the image from light engine to human eye^209^. A large Etendue benefits from the exit pupil expansion (EPE) process. In practicality, the FoV is still a bottleneck, and the EPE is expected to be 2D in consideration of eyebox expansion in both horizontal and vertical directions. The practical FoV is jointly determined by the refractive index (n_g_) of the waveguides and the angular bandwidth provided by the couplers. A large n_g_ waveguide with wide angular response of the couplers is conducive to expand the FoV.

Currently, surface relief gratings (SRGs) are dominant couplers in waveguide AR products. SRGs have a large index modulation to produce wide angular response and can achieve a uniform EPE, although the design is somewhat sophisticated^210^. As the input coupler, both transmissive and reflective SRGs can be considered (Fig. 14a). For the output coupler, a reflective type with suppressed transmission orders is preferred^211^. Compared to SRGs, PVGs exhibit a smaller index difference, but spatially variant LC orientations, strong circularly polarized responses, thin-film form factor, and easy fabrication process. To obtain a uniform light output, a polarization management layer consisting of an LC layer with spatially varying orientations can be utilized^212^. It also offers an additional degree of freedom to control the polarization state of the TIR light.

In the following, we will review some methods employing PVGs as the couplers in the waveguides. To display full-color images, it is feasible to stack R/G/B PVGs as the in- and out-couplers on one waveguide. Weng et al.^213^ proposed a dual-waveguide structure for a full-color AR. The blue and green PVGs are laminated together on one waveguide, and the red PVG is put solely on the other waveguide (Fig. 14b). Aside from full-color images, for practical applications, it is necessary to take the PVG’s angular response into account to analyze the FoV, especially focusing on the angular bandwidth with 0^o^ as the symmetric center for the in- and out-couplers. The analysis with simulation parameters is listed in Supplementary S1. The angular response of a PVG can be adjusted by two methods: changing the LC birefringence and stacking several layers with different Bragg pitches^208^. Recently, Gu et al.^214^ experimentally demonstrated this approach based on stacking two PVGs with opposite polarization responses to extend the angular response at the target wavelength, thereby enlarged the observed FoV (Fig. 14c).

In addition to enlarging the angular response, in a waveguide AR, a uniform eyebox is also critical. Generally, there are two methods to create a uniform eyebox. The first solution is to manipulate the local efficiency at different out-coupling regions on the PVG^20^. Nevertheless, the gradient efficiency of PVG (out-coupler) will cause uneven ambient light transmittance to the user’s eye. Another solution is to use a PVG with uniform efficiency but attach an LC-based polarization modulation layer (PML) to the bottom of the waveguide^212^. By modulating the azimuthal angles of the LC directors in the PML, the phase control and thus the polarization control can be achieved. As a result, the output light intensity could be uniform because the reflection efficiency of the PVG is dependent on the light polarization.

A reflective LC lens can be obtained by introducing a parabolically varying phase to the CLC reflector. The lens phase profile can be superposed onto either planar-CLC or slanted-PVG pattern to form an on-axis or off-axis CLC lens (Fig. 12c, d). The CLC lens still follows the polarization selectivity rule as the CLC reflector but exhibits opposite phase profiles when the incident light impinges from different sides^215,216^. In other words, the incident light with the target polarization can be converged or diverged, depending on which side the beam enters. Leveraging the polarization selectivity of CLC, multiple CLC films can be stacked together to perform different functionalities for a wide range of applications.

**Fig. 15:**
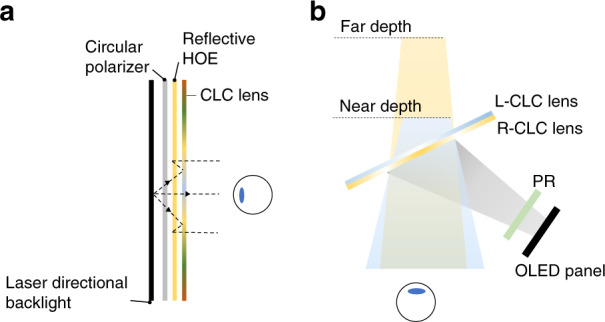
Schemes for on-axis CLC lens and applications in AR/VR. **a** Ultra-compact Pancake VR display with only HOEs. **b** Dual-depth AR display. PR: polarization rotator. L-CLC lens: LCP response; R-CLC lens: RCP response

For a VR system, the CLC reflector in pancake design can be further replaced by an on-axis CLC lens. Such a CLC lens provides more degrees of freedom for optimizing the optical aberrations^217^. The combination of refractive and diffractive elements suppresses the chromatic aberrations due to their opposite dispersion behaviors, like the result shown in Fig. 11c. The pancake structure can be further evolved to a more compact form factor, if all the refractive optical elements are replaced by the HOEs^51^ (Fig. 15a). To accommodate the angular selectivity and wavelength selectivity of the HOEs, a monochromatic directional backlight (e.g., 532 nm laser source) is preferred as the light engine. Besides, the on-axis CLC lenses can be implemented in projection AR systems (Fig. 1b) to serve as optical combiners. Li et al.^218^ proposed a dual-depth AR system^219,220^ by tilting the CLC lens at 22.5° relative to the eye pupil (Fig. 15d). Since the polarization dependent CLC lenses could distinguish two circular polarizations and apply different diopters, two image depths can be obtained.

**Fig. 16:**
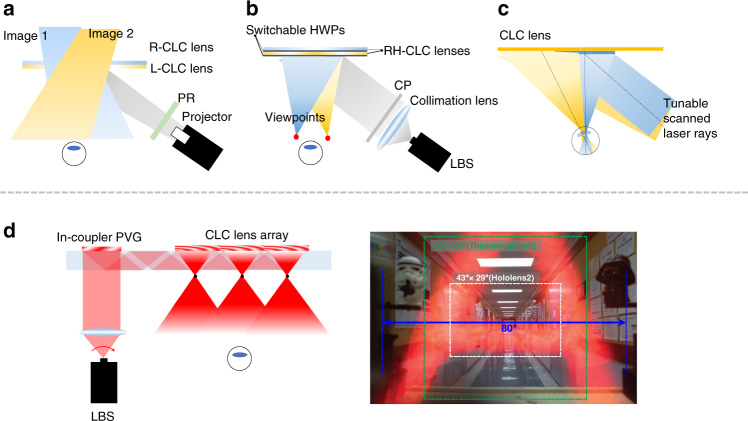
Schemes for off-axis CLC lens and applications in AR system. **a** Doubled FoV AR display; **b** Pupil steering Maxwellian AR display; **c** Gaze-matched pupil steering Maxwellian display. **d** Scanning waveguide AR display. LBS: laser beam scanning. Reproduced from **d** ref. ^229^ with permission from The Optical Society

For off-axis CLC lenses, Yin et al.^221^ proposed a two off-axis CLC lenses based multiplexing system for projection AR systems (Fig. 1b) to expand the FoV. As shown in Fig. 16a, each lens responds to a different circular polarization and works for a specific FoV. By combining the two views, an expanded tiled FoV can be presented in front of the observer^222^. It should be mentioned that the image aberration could be caused by the dispersion of diffractive elements and large off-axis angle^223^, especially with a wideband light source. To mitigate these aberrations, digital compensation or a light source with narrow bandwidth can be adopted. The off-axis CLC lenses can also serve as the diffractive couplers in Maxwellian displays^224–226^. Maxwellian view has a tiny eyebox, which is usually assumed to be the pupil size (3–4 mm). The implementation of CLC lenses could perform pupil duplication or pupil steering to expand the eyebox. Pupil duplication refers to the scenario in that multiple viewpoints are formed simultaneously to cover a larger eyebox. The light intensity in each viewpoint is determined by the total light intensity divided by the viewpoint numbers. For pupil steering, the viewpoint position is timely shifted in response to the pupil location detected by the eye-tracking technique. This approach offers an excellent optical efficiency. Xiong et al.^224,227^ proposed a pupil steering structure by using Maxwellian system with off-axis CLC lenses (Fig. 16b). Each CLC lens is coupled with a switchable HWP. In the voltage-on state, the HWP converts the LCP light into RCP light, which is reflected by the first CLC lens and steered into the first viewpoint. As the pupil moves to a second viewpoint, the first HWP is turned off and the second HWP is switched on. As a result, the second CLC lens reflects the image toward the pupil. More viewpoints can be accommodated by stacking more CLC lenses and using ultrathin (~100 μm) glass substrates to keep the package compact and lightweight.

Apart from generating or switching between several viewpoints, a gaze-matched function can also be realized to accommodate the eye rotation in a Maxwellian system. In the scenario of gaze-matching, the exit pupil should be aligned with the ocular pupil. In other words, the chief ray should match with the eye gazing direction. To meet this requirement, the incident light angle on the imaging couplers should be modulated in real time. Kim et al.^228^ proposed a mechanical shifter to shift the horizontal position of the imaging coupler. This approach can directly achieve gaze-matching; however, the mechanical shifter increases the complexity and weight of the system. The aberration will also arise when the same holographic lens coupler is shifted, since the probing wave on the holographic lens coupler fails to match the wavefront of the reference wave used in the exposure process. Zou et al.^150^ developed a gaze-matched Maxwellian system by employing multiple off-axis CLC lenses with different patterns to accommodate different incident light directions. As shown in Fig. 16c, three lenses are designed for three exit pupils to align with the ocular pupil positions when the eye rotates. Since each lens can be specifically designed for one incident angle, the aberration will be negligible. However, this approach could become bulkier if more viewpoints are required to cover a large range of eye rotation. To make it reasonably practical, some compromise can be made between the viewpoint numbers and the degree of gaze-matching. Although the Maxwellian display is limited to a tiny eyebox, recalling that the waveguide framework can support a large eyebox. By combining these two advantages together, Xiong et al.^229^ proposed a scanning waveguide display to achieve a wide horizontal FoV (~80°) by deploying an off-axis CLC lens array as the out-coupler (Fig. 16d). The collimating light is outcoupled by the off-axis CLC lens array and then forms multiple viewpoints. In this framework, the FoV is no longer constrained by the refractive index of the waveguide but solely decided by the *f*-number of each lens element. Because of the robust CLC alignment, the off-axis CLC lens can reach a small *f*-number (~0.6).

## Conclusion and outlook

AR/VR technologies combined with advanced LC devices have emerged as a significant new direction for novel displays and information platforms, with potential for a wide range of applications. To enable a compact form factor and high image quality in wearable AR/VR headsets, advanced LC devices play important roles, ranging from light engines to various functional optics. In this review, we divide these advanced LC devices into three categories: high-dynamic-range LCDs, LCoS including amplitude and phase modulators, and planar optics. Although LCDs for VR headsets suffer from low transmittance issues caused by small aperture ratios and disclination lines blocking light, achieving a high-performing VR that is compatible with directional backlight and spatial patterned modulator techniques represents a promising solution. At the same time, LCoS SLMs offer unrivaled phase modulation to realize holographic views beyond conventional displays. Further advancements in device engineering and fabrication processes are expected to improve the performance of LCDs and LCoS SLMs in AR/VR applications. Emerging LC planar optics provide excellent optical properties with an ultrathin form factor and high efficiency. These advanced LC-based devices play pivotal roles for systematically improving the image quality and form factor of the AR/VR displays.

## Supplementary information

Supplementary Information

## Data Availability

All data needed to evaluate the conclusions in the paper are present in the paper. Additional data related to this paper may be requested from the authors.
