# Supplementary material for: Advanced liquid crystal devices for augmented reality and virtual reality displays: principles and applications
Source: Light Sci Appl. 2022 May 30;11:161. doi: 10.1038/s41377-022-00851-3 (PMC9151772; doi:10.1038/s41377-022-00851-3)
Supplement: Supplementary file 1 — Supplementary Information [file 41377_2022_851_MOESM1_ESM.docx]

**Supplementary Information for Advanced liquid crystal devices for augmented reality and virtual reality displays: Principles and Applications**

*Kun Yin, En-Lin Hsiang, Junyu Zou, Yannanqi Li, Zhiyong Yang, Qian Yang, Po-Cheng Lai, Chih-Lung Lin, and Shin-Tson Wu^*^*

*College of Optics and Photonics, University of Central Florida, Orlando, FL 32816, USA*

*Corresponding author: [swu@creol.ucf.edu](mailto:swu@creol.ucf.edu)

_____________________________________________________________________________

**S1 PVG calculation parameters**

To display full-color images, it is feasible to stack R/G/B PVGs as the in- and out-couplers on one waveguide. However, it is indispensable to ensure the period-matching for all gratings to avoid the crosstalk issue. In this circumstance, the effective FoV significantly shrinks since each color possesses a different FoV. Figure S1 depicts the FoV distribution of RGB light by assigning the matched period to all PVGs. The specific parameters are listed in Table S1.


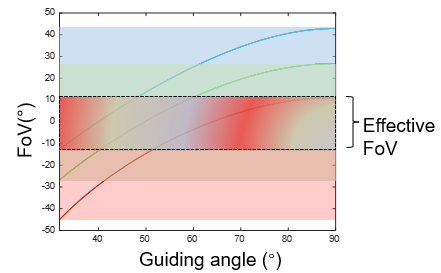


**Fig. S1 PVG FoV simulation results.** Plot of FoV distribution of RGB light. All RGB PVGs are assigned to the same horizontal period.

**Table S1: The parameters used in PVG calculations**

| Blue wavelength (nm) | Green wavelength (nm) | Red wavelength (nm) | n_g_ | Critical angle (^o^) | Maximum guiding angle (^o^) | Grating period (nm) |
| --- | --- | --- | --- | --- | --- | --- |
| 450 | 535 | 630 | 1.9 | 31.7 | 90 | 368.96 |
